# Optimization of Extraction Parameters for Enhanced Production of Ovotransferrin from Egg White for Antimicrobial Applications

**DOI:** 10.1155/2015/934512

**Published:** 2015-11-10

**Authors:** Eyad M. A. Alshammari, Saif Khan, Arshad Jawed, Mohd Adnan, Mahvish Khan, Gowher Nabi, Mohtashim Lohani, Shafiul Haque

**Affiliations:** ^1^Department of Clinical Nutrition, College of Applied Medical Sciences, University of Ha'il, Ha'il 2440, Saudi Arabia; ^2^Research and Scientific Studies Unit, College of Nursing & Allied Health Sciences, Jazan University, Jazan 45142, Saudi Arabia; ^3^Department of Genetics, College of Applied Medical Sciences, Jazan University, Jazan 45142, Saudi Arabia; ^4^Department of Biosciences, Integral University, Lucknow, Uttar Pradesh 226026, India; ^5^Department of Biosciences, Jamia Millia Islamia, New Delhi 110025, India

## Abstract

Ovotransferrin is the second most abundant protein (~12-13% of the total egg protein) in egg white after ovalbumin. Ovotransferrin is a potent natural antimicrobial agent as it possesses antibacterial, antifungal, and antiviral properties and is also the major metal binding protein found in egg, which makes it an industrially important protein. Ovotransferrin was extracted from egg white using its metal (iron) binding properties. In the present study, eggs from two different sources were used (fresh local eggs from domestic household source and poultry eggs from shops) to compare the results and Response Surface Methodology was used for the experiment design and data analysis. The following extraction conditions were optimized so as to maximize the yield of ovotransferrin from egg white: ethanol % (v/v) and pH and volume (mL) of 25 mM FeCl_3_/50 mL of egg white. A maximum yield of ~85 ± 2.5% was obtained near the optimum extraction conditions. The yield was calculated based on the theoretical value (934 mg) of ovotransferrin in 100 mL of 1.5x diluted egg white solution. Our results suggest that efficient downstream processing may reduce the cost of overall production process of this promising enzyme, making it a natural and cost-effective alternative to the existing chemically synthesized antimicrobial agents.

## 1. Introduction

Transferrins are iron binding proteins that control the level of free iron in biological fluids, especially in the blood plasma [1]. Transferrins can be divided into 4 categories according to their occurrence, namely, (a) serum transferrins (present in plasma), (b) lactoferrins (present in milk and other secretions in mammals), (c) melanotransferrins (expressed on cell surface of melanoma cells), and (d) ovotransferrin (found in egg white). All of these proteins have iron binding ability that endows them with antimicrobial activity by rendering iron unavailable for the growth of microorganisms. It has been recently discovered that ovotransferrin interacts directly and binds with the surface proteins present on bacterial cells [2, 3].

Ovotransferrin is a single chain glycopeptide having a molecular weight of 77.9 kDa (total of 686 amino acids) with isoelectric point (pI) of 6.0 that makes it acidic in nature. It is made up of two homologous halves each possessing binding site for iron [4–8]. It also exists in two forms in nature, that is, apo-ovotransferrin (deprived of iron) and holo-ovotransferrin (saturated with iron) [9]. Holo-ovotransferrin is relatively more stable as apo-ovotransferrin is easily destroyed by physical and chemical treatments. Higher stability and metal binding properties were utilized to selectively isolate ovotransferrin [10]. It is the second most abundant protein in egg white after ovalbumin, present at a concentration of 12-13% (v/v) in egg white protein [6]. It displays an array of bioactivities and it is considered as a potent candidate for being utilized as a natural antimicrobial agent. Ovotransferrin also bears close resemblance to lactoferrin, an important component of innate immune system and a natural immune modulator. Therefore, if produced at large scale, it can be also used for treating inflammation and cancer and as an iron supplementing agent in humans, as with lactoferrin [11, 12].

The success of any bioprocess depends on the purity and the yield achieved with strong consideration of process economics. Compared to the studies on other transferrin molecules, research on ovotransferrin is limited. Since ovotransferrin requires posttranslational modifications in the form of glycosylation, it is not feasible to be efficiently cloned in commonly used bacterial hosts. The most frequently used methods of extracting ovotransferrin from avian eggs include precipitation under low pH, in presence of ammonium sulphate or ~50% (v/v) ethanol at pH of 6–9 [10, 11]. Chromatographic methods have been also explored for the improvement in the purity as these methods are easy to implement and scale up [7, 12]. Cation exchange chromatography with CM cellulose was used by Rhodes et al., but it resulted in contamination by globulins [13]. The separation method was further improved by Azari and Baugh in 1967 by the addition of precipitation and crystallization step leading to higher purity in the final product [7]. Subsequent studies by various groups concentrated on DEAE-Sepharose resulted in higher purity of 94–98% as indicated by SDS-PAGE analysis [14]; still, no process has been found to be feasible for large scale process development. Ovotransferrin recently has been purified with 80% purity by various groups [15–17], but in all the cases either the process is cumbersome or recoveries are compromised making the process impractical to be scaled up at industrial level.

In the present study, we optimized the productivity of ovotransferrin from domestic household and poultry eggs. Our aim was to achieve substantial improvement in the yield of purified ovotransferrin resulting in a process that is commercially feasible and can be scaled up to industrial levels for pharmaceutical applications. We employed Response Surface Methodology (RSM) to perform the experiments for the optimization studies rather than the conventional one-factor-at-a-time (OFAT) approach. Statistical optimization methods are more accurate and less time consuming and take into account the complicated interactions between multiple process parameters that affect the outcome of the experiment; in our case, it affects overall yield and purity of ovotransferrin. RSM generates a robust statistical model using full or partial factorial design. The generated mathematical relationship can be used to predict the experimental responses taking into account the interactions between the process components.

**Figure 1:**
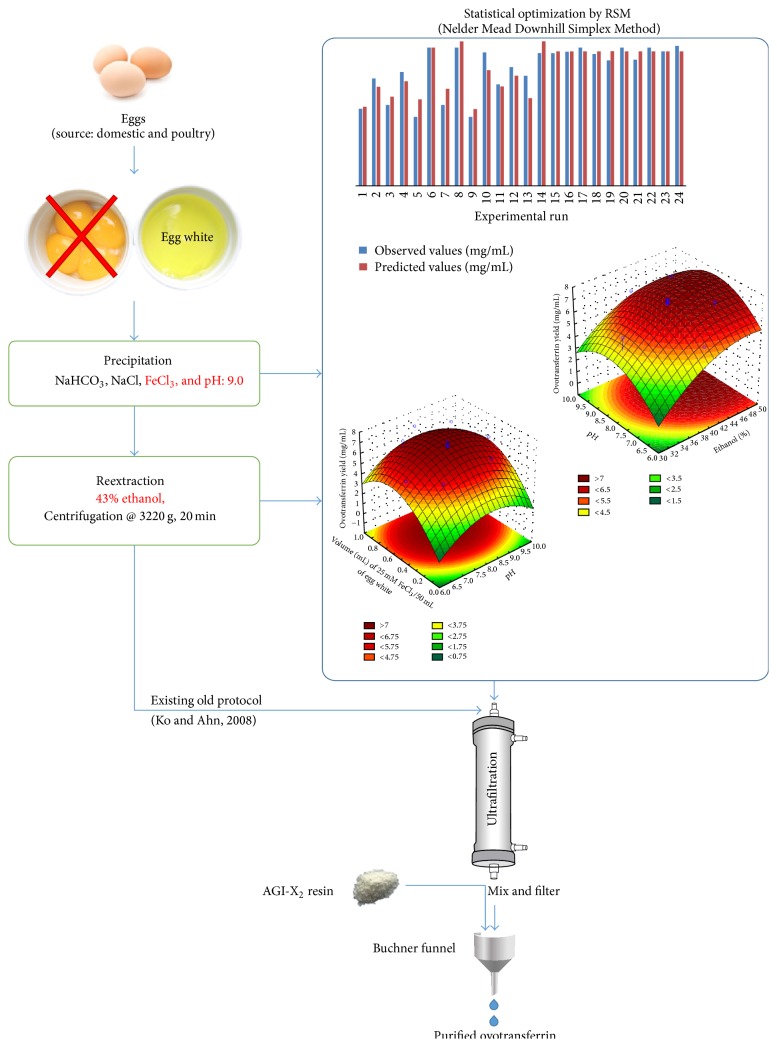
Graphical representation of the entire extraction optimization process of ovotransferrin.

There are several techniques available for the optimization of mathematical models explaining the relationship between the cause and the effect [18, 19]. In the present study, the response surface equation (obtained by applying RSM) explains the relationship between the extraction conditions and the enzyme yield. This response was then optimized via Nelder Mead Downhill Simplex (NMDS) optimization technique. NMDS is a single-objective optimization approach for searching the space of n-dimensional real vectors [20]. Earlier reports have shown that NMDS has been successfully applied for the modelling and optimization of a variety of chemical and biological processes [21, 22]. Since it only uses the values of the objective functions without any derivative information (explicit or implicit), it falls into the general class of direct search methods [23, 24]. The graphical representation of the entire process has been shown in the form of graphical abstract as Figure 1.

## 2. Materials and Methods

### 2.1. Chemicals and Biochemicals

All chemicals/biochemicals used in this study were procured from Bio-Rad Laboratories (USA), Sigma Aldrich (USA), BDH Limited (India), Hi-Media Laboratories (India), and RFCL Limited (India). The eggs used in the study were obtained from locally reared (household) hen and poultry eggs (Al-Watania, Saudi Arabia) were procured from local grocery stores well within the expiry date. The SDS PAGE protein ladder (Prism Ultra Protein Ladder) was purchased from Abcam, Singapore.

### 2.2. Extraction of Ovotransferrin

Ovotransferrin was extracted from eggs procured from local and poultry sources as described by Ko and Ahn [25]. Briefly, egg white was separated from the eggs and diluted with the same volume of distilled water. The pH of 1.5x diluted egg white (household and poultry source) solution was adjusted to 8.0 with 50 mM citric acid solution. Since ovotransferrin binds with iron (III), it was added in the form of FeCl_3_ to selectively stabilize ovotransferrin in the solution. 0.5 M of ferric iron chloride (1.6 mL of 0.5 M FeCl_3_·6H_2_O solution per liter egg white solution) was used. The iron-added egg white solution was homogenized and mixed using a magnetic stirrer for 2 min. After standing at room temperature for 1 h, 100% cold ethanol (43% v/v final ethanol concentration) was added to precipitate other proteins including ovalbumin, whereas ovotransferrin remains in solution. Holo-ovotransferrin in the supernatant was separated from the precipitated egg white proteins by centrifugation at 3,220 ×g for 40 min. The precipitate was reextracted with the same concentration of ethanol and centrifuged at 3,220 ×g for 40 min. The supernatants were pooled and filtered through filter paper to remove the floating materials. After filtering, cold ethanol (100%) was slowly added to the supernatant to the final ethanol concentration of 63% (v/v) to precipitate iron-bound ovotransferrin. The precipitated holo-ovotransferrin was collected after centrifugation at 3,220 ×g for 20 min and resuspended in water.

### 2.3. Design of Experiments

MATLAB and Statistica software programs were used for statistical/mathematical calculations and analysis of the data. Design of experiments was planned with three variables,* namely,* ethanol (*x*_1_), pH (*x*_2_), and 25 mM FeCl_3_ (*x*_3_) as shown in Table 1.  Table 2 demonstrates the actual experiments performed according to the Central Composite Design (CCD) given by Box and Wilson [26]; each row of Table 2 corresponds to a single experiment, where the extraction conditions were changed accordingly. The central values (zero level) were chosen at 40% (v/v) for ethanol and 1 mL for 25 mM FeCl_3_/100 mL of egg white and pH was selected at a central level of 8 for the CCD. A total of 24 experiments that included eight cube points (runs 1–8), six star points (runs 9–14), and ten replicas of the central points (runs 15–24) were required to fit the second-order polynomial model. Values of the test variables [ethanol % (v/v) extraction and pH and volume (mL) of 25 mM FeCl_3_/100 mL of egg white] were kept according to CCD. Protein estimation was done for each row of CCD (Table 2). The purity of the protein yield was tested by SDS page as discussed below. The quantification of ovotransferrin was performed densitometrically and spectrophotometrically.

### 2.4. NMDS Optimization

NMDS was implemented on MATLAB platform using the following input parameters:

 
*‘Largescale', ‘off', ‘Simplex', ‘on', ‘TolFun', 1.0e − 06, ‘MaxIter', 10000, ‘MaxFunEvals', 60000, ‘Display', *and* ‘Iter'*.

### 2.5. Size Exclusion Chromatography

Size exclusion chromatography experiments were performed on Superdex 200 column (manufacturer's exclusion limit 600 kDa for proteins) connected with an automated AKTA prime FPLC apparatus (Amersham Pharmacia Biotech, Sweden). The column was equilibrated and run with 20 mM KPB (pH 8). Five hundred microliters of the sample were injected into the column. The column was calibrated with the low molecular weight gel filtration calibration kit (Amersham Biosciences). Elution profiles were recorded at 215 nm under a constant flow rate of 0.3 mL/min. Size exclusion chromatography was performed on the finally extracted ovotransferrin samples before and after 5x dilution in order to determine their purity.

### 2.6. Quantification of Ovotransferrin

The quantification of ovotransferrin was done spectrophotometrically by biuret test of proteins [27]. Since the concentrations of the samples obtained from CCD may be beyond the biuret test range, hence all the samples obtained from CCD runs were 5x diluted. Bovine serum albumin (BSA; Sigma-Aldrich, USA) was used as standard. The purity of the 5x diluted samples was also tested on sodium dodecyl sulfate polyacrylamide gel electrophoresis (SDS-PAGE) by loading equal volumes (1 *μ*L) of the extracted samples onto each lane from each run (Run numbers 1–16). SDS PAGE was performed at 10% acrylamide concentration followed by overnight staining with Coomassie brilliant blue [28].

## 3. Results

### 3.1. Ovotransferrin Extraction

**Figure 2:**
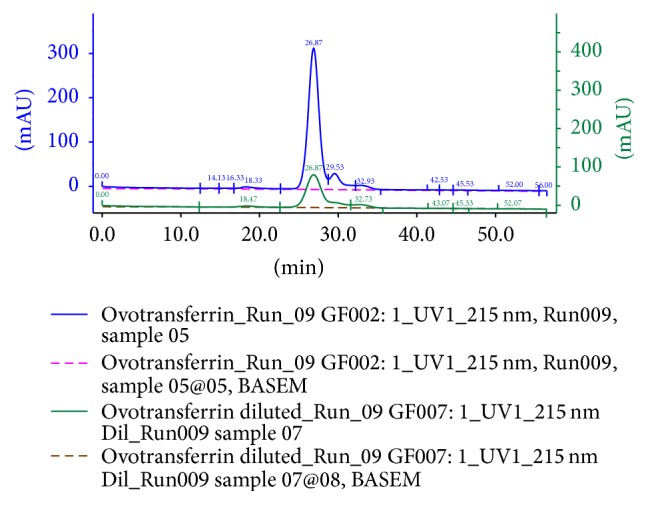
Size exclusion chromatography: stacked chromatogram of undiluted and diluted (5x) samples of extracted ovotransferrin.

The study was initiated with the determination of the ovotransferrin concentration under the following conditions: 40% (v/v) ethanol in a two-step extraction process, pH 8, and 0.5 mL 25 mM FeCl_3_/50 mL of egg white for both egg sources (domestic household and poultry). The concentration of ovotransferrin extracted from poultry eggs was found to be higher at 6.07 mg/mL as compared to 5.2 mg/mL for domestic household eggs. Higher ovotransferrin content can be attributed to various optimized and nutritionally rich feeds given to the hens in poultry as compared to almost no special feed given to the hens under domestic household conditions. As the yield of ovotransferrin was found to be better in poultry eggs, the experiments were continued with the same. The purity of the extracted ovotransferrin was determined by size exclusion chromatography. The stacked chromatogram shows the profile for both undiluted and diluted (5x) samples of extracted ovotransferrin (Figure 2). In the undiluted profile, the contamination of ovoalbumin is clearly visible. The percentage purity of ovotransferrin was determined from the area under the peak calculation and it was found to be ~81%. However, in the diluted ovotransferrin profile, there is hardly any ovoalbumin visible (Figure 2).

**Figure 3:**
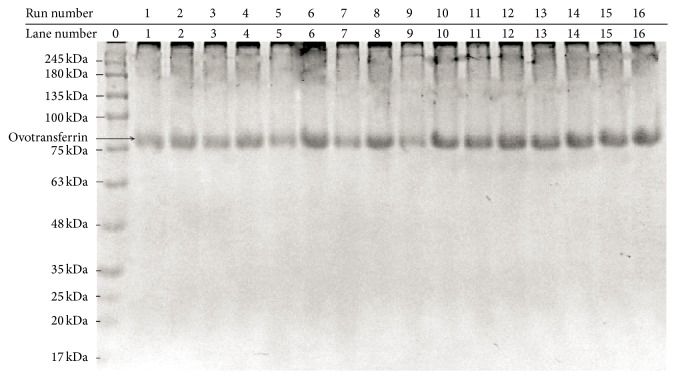
SDS-PAGE analysis of extracted ovotransferrin as per the CCD runs.

The statistical optimization of ovotransferrin extraction was then performed on poultry eggs as per the CCD given in Table 2. The results of the CCD experiment are also included in the same table (kindly refer to Table 2). The 5x diluted extracted ovotransferrin samples from the CCD were subjected to biuret test. The concentration of ovotransferrin was finally calculated taking into account both the extent of dilution (5x) and the degree of purity (81%). The final values are reported in Table 2 against each run. The 5x diluted extracted ovotransferrin samples from the initial 16 runs were subjected to SDS-PAGE. Almost pure ovotransferrin bands were visible at this degree of dilution (Figure 3).

### 3.2. Analysis of Response Surface Model

**Figure 4:**
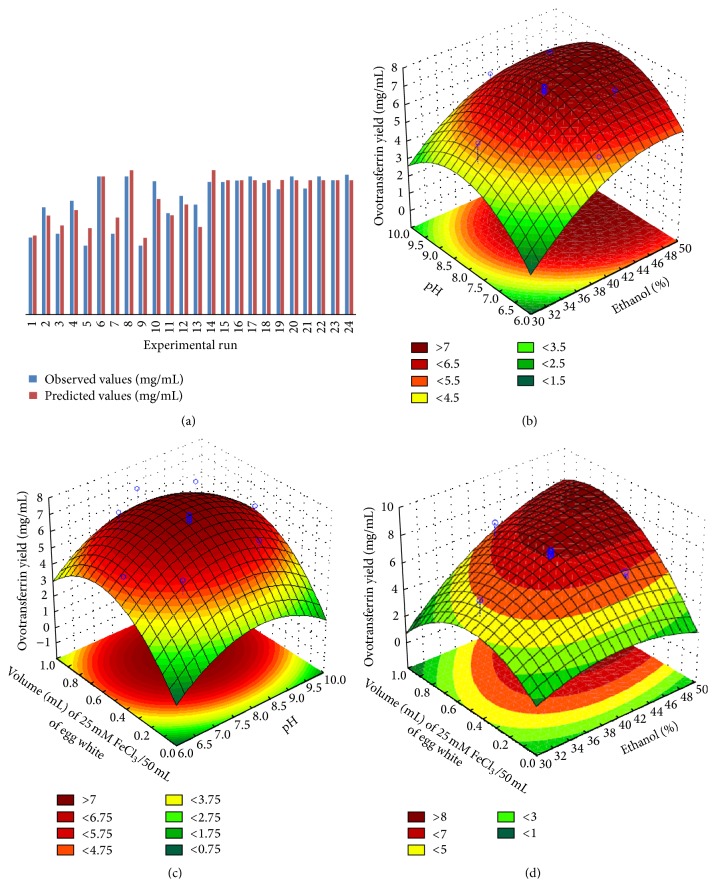
(a) Predicted versus actual ovotransferrin yield. (b) Fitted response surface showing the combined effect of ethanol (%; *x*_1_) and pH (*x*_2_) on ovotransferrin yield. (c) Fitted response surface showing the combined effect of volume of FeCl_3_ (*x*_3_) and pH (*x*_2_) on ovotransferrin yield. (d) Fitted response surface showing the combined effect of volume of FeCl_3_ (*x*_3_) and ethanol (%; *x*_1_) on ovotransferrin yield.

The result of the second-order response surface model fitting in the form of analysis of variance (ANOVA) is shown in Table 3. The very low probability value (*p* value ≪ 0.05) demonstrated a very high significance for the regression model as predicted by the CCD. The goodness of fit of the model was checked by the determination coefficient (*R* ^2^). In this case, the value of the determination coefficient (*R* ^2^ = 0.94553) indicated that the model did not explain only 5.9% of the total variations. The value of the adjusted determination coefficient (Adj. *R* ^2^ = 0.89623) was also very high, which indicated a high significance of the model. A high value of the correlation coefficient (*R* = 0.89623) signified an excellent correlation between the independent variables. In Figure 4(a), each of the observed values for the yield was compared with the predicted values. Ovotransferrin yield predicted by the second-order polynomial response equation (1) is quite close to the experimentally observed values. All of the above considerations suggested an excellent adequacy of the polynomial regression model. The amount of ovotransferrin obtained was subjected to SDS-PAGE (Figure 3) and quantified against each run as per the CCD. Biuret test was also successfully done to quantify the amount of ovotransferrin.

### 3.3. Response Equation

Mathematical packages MATLAB and Statistica were used to perform the regression and the graphical analysis of the results obtained from the CCD experiment. A second-order polynomial response equation (of the form given below) comprising linear, quadratic, and interaction terms was obtained:

(1) $$\begin{array}{c} Y=b_{0}+\sum b_{i}x_{i}+\sum b_{i}^{2}x_{i}^{2}+\sum b_{i,j}x_{i}x_{j}. \end{array}$$

### 3.4. Model Coefficients and Their Significance

The significance of each regression coefficient was determined by Student's *t*-test, *t*-values, and *p* values. The regression coefficients, *t*-values and *p* values for single and each interaction term, have been listed in Table 4. All the second-order or quadratic main effects of the extraction variables were found to be significant; the negative values of all the quadratic main effects suggested that all the constituents had an adverse effect at experimental outcomes, at higher concentration, which was overcome by higher positive first-order main effects. The fact that all the quadratic terms were significant suggested considerable curvature in the model. A positive significant interaction was observed (*p* = 0.05094 < 0.01) between ethanol % (v/v) in the first and the second extraction and the volume (mL) of 25 mM FeCl_3_/50 mL of egg white. However, the interaction of ethanol % (v/v) in the first and the second extraction and the pH (*p* = 0.8 ≫ 0.01) and the interaction of the volume (mL) of 25 mM FeCl_3_/50 mL of egg white and the pH (*p* = 0.97 ≫ 0.01) were not found significant. A high negative quadratic main effect (−11.28) of the volume (mL) of 25 mM FeCl_3_/50 mL of egg white on ovotransferrin yield shows a high negative effect by the addition of large volumes of 25 mM FeCl_3_/50 mL of egg white. A positive linear effect of the pH (9.0) shows an enhancing effect of pH on ovotransferrin yield, whereas a negative quadratic main effect of the pH balances the positive enhancing effect at higher pH values.

### 3.5. Response Equations and Optimum Values

The application of RSM yielded the following regression equation, which shows an empirical relationship between the ovotransferrin yield and the test variables [ethanol % (v/v) in the first and the second extraction, pH, and volume (mL) of 25 mM FeCl_3_/50 mL of egg white]:

(2) Y=−56.7598+1.1429x1+9.0029x2−0.1206x3−0.011x12−0.5214x22−11.2827x32−0.0115x1x2+0.3400x1x3+0.03001x2x3,

where *Y* is the response for ovotransferrin yield in mg/mL and *x*_1_, *x*_2_, and *x*_3_ are the values of the test variables [ethanol % (v/v): *x*_1_, pH: *x*_2_, and volume (mL) of 25 mM FeCl_3_/50 mL of egg white: *x*_3_].

Response surface plots [Figures 4(b)–4(d)] as a function of two variables at a time, maintaining the third variable at a fixed level (central values of CCD), are drawn from the predictions of the response equation (2). These plots present an easy way to visualize the optimum region of the extraction variables, where the maximum ovotransferrin yield is obtained. These plots are also helpful in understanding the interaction effect of the extraction conditions on ovotransferrin yield. Figures 4(b)–4(d) show all the possible interaction response surfaces. Interesting conclusions regarding the interactive behavior of ethanol % (v/v) in the extraction: *x*_1_, pH: *x*_2_, and volume (mL) of 25 mM FeCl_3_/50 mL of egg white: *x*_3_ can be drawn by observing the localization of high and low yield regions on the response surfaces. Dark red regions correspond to high yield regions while green regions correspond to very low yields of ovotransferrin. All the response surfaces were drawn by plotting the selected two variables while keeping the third variable at the center of the CCD design (Table 2).

The response surface graph (Figure 4(b)) shows the combined effect of ethanol % (v/v) on extraction (*x*_1_) and pH (*x*_2_) on ovotransferrin yield. It can be observed that a combination of very low pH and very low ethanol concentration on extraction results in a very poor yield of ovotransferrin, whereas the ovotransferrin yield increases with the increase in both pH and ethanol % (v/v) in subsequent extractions. This is due to the strong positive effects of these extraction conditions and weak insignificant negative effects. The optimum values appear to lie towards the higher extreme of pH and ethanol % (v/v) in the extraction process. Ovotransferrin yield decreases drastically towards lower levels of ethanol % (v/v) even when pH values are taken to higher levels. There is enough curvature in the response surface and the optimum values are observed to lie within the range considered.

The fitted response surface shows the combined effect of pH (*x*_2_) and volume (mL) of 25 mM FeCl_3_/50 mL of egg white (*x*_3_) on ovotransferrin yield as depicted in Figure 4(c). It can be observed that both higher and lower values of pH (*x*_2_) and volume (mL) of 25 mM FeCl_3_/50 mL of egg white result in poor ovotransferrin yield. The most efficient extraction conditions lay near the central values of these two parameters considered.

The response surface showing the combined effect of ethanol % (v/v) in extraction (*x*_1_) and volume (mL) of 25 mM FeCl_3_/50 mL of egg white (*x*_3_) on ovotransferrin yield is shown in Figure 4(d). It can be observed that lower extremities of these two parameters result in very inefficient extraction even though a combination of higher *x*_3_ and lower *x*_1_ resulted in poor extraction of ovotransferrin. Maximum extraction of ovotransferrin was observed somewhere near the higher edge of *x*_3_ and *x*_1_ combination. Assuming the initial 1.7x diluted egg white contains 9.45 mg/mL of ovotransferrin, the expected yield (around the optimum area) is ~85 ± 2.5%. The yield was calculated based on the theoretical value (934 mg) of ovotransferrin present in 100 mL of 1.5x diluted egg white solution. The extracted ovotransferrin was subjected to size exclusion chromatography in order to check the purity and the yield of ovotransferrin obtained from the solvent based extraction process. The chromatographic profile showed single major peak indicating pure ovotransferrin with limited impurities.

### 3.6. Optimum Extraction Conditions

**Figure 5:**
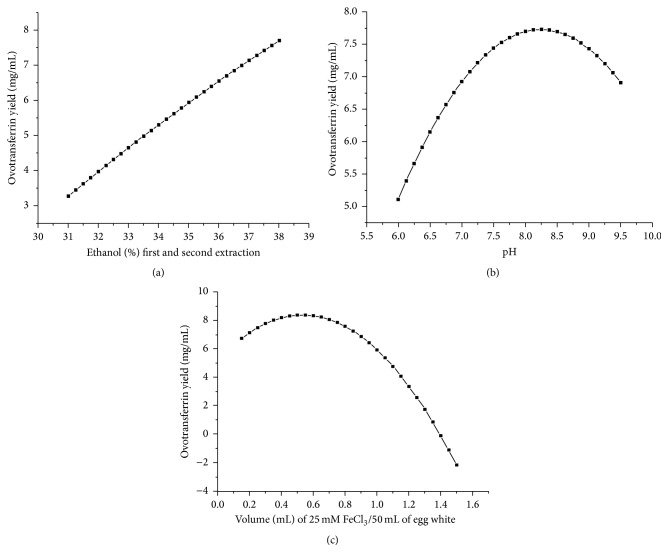
Individual effect of extraction conditions on ovotransferrin yield: ovotransferrin yield plotted as a response obtained by varying individual extraction conditions keeping the other two at central values of the CCD: (a) ethanol % (v/v) in first and second extraction, (b) pH, and (c) volume (mL) of 25 mM FeCl_3_/50 mL of egg white.

The following optimum extraction conditions were obtained after NMDS optimization of the response equation: ethanol % (v/v) in the first and the second extraction: 47.87 mL; pH: 8.14; volume (mL) of 25 mM FeCl_3_/50 mL of egg white: 0.73 mL. The predicted ovotransferrin yield was 8.06 mg/mL and the experimentally observed average ovotransferrin yield at optimum points was 7.8 mg/mL. The individual effect of extraction conditions on ovotransferrin yield was determined by the response equation (2). The ovotransferrin yield was plotted as a response obtained by varying the individual extraction conditions,* namely,* ethanol % (v/v) in the first and the second extraction (Figure 5(a)), the pH (Figure 5(b)), and the volume (mL) of 25 mM FeCl_3_/50 mL of egg white (Figure 5(c)), respectively, while keeping the remaining two at fixed respective central values of the CCD.

Ethanol % (v/v) in the first and the second extraction appears to have an almost linear effect on ovotransferrin yield. However, the pH has a parabolic effect on ovotransferrin yield with the maximum at somewhere ~pH 8. This may be possibly due to unfavorable conformational changes introduced in the protein structure because of highly basic environment at higher pH.

Since loss in protein conformation adversely affects its iron binding properties, any loss of protein conformation is reflected as reduced protein yield. From the pH profile, it appears that the protein maintains its conformation in the acidic region considered (5.5–7) in this study.

The fact is supported by acid unfolding analysis of this protein, where it loses its conformation only below pH 4 [29]. This confirms decreased iron binding at higher pH. Increasing the volume (mL) of 25 mM FeCl_3_/50 mL of egg white above 0.55 mL has negative effect on ovotransferrin yield. Interestingly, increasing FeCl_3_ increases the ionic interaction at higher concentrations as these ionic interactions may disturb the conformation and favor the binding of ferric ions with the protein thereby decreasing ovotransferrin yield.

## 4. Discussion

Ovotransferrin is a major egg white protein that acts as a second line of defense against invading microbes and can be used as a natural antimicrobial agent. The multifunctional properties of ovotransferrin,* namely*, antimicrobial, antifungal, and antiviral activities, make it a potent candidate to be researched extensively. Its role as natural immune modulator and promising role in cancer therapy makes the molecule exciting for commercial applications. This study brings up several key findings for efficient extraction of ovotransferrin from eggs obtained from domestic household and commercial poultry farm sources. The eggs obtained from the poultry showed comparatively higher ovotransferrin concentration than the eggs obtained from the domestic household source. Higher ovotransferrin content can be attributed to balanced and controlled feed provided in commercial poultry farms. Poultry feeds have high content of proteinaceous substances that produce bigger chicken with more flesh content. Therefore, eggs from poultry sources were selected for subsequent experiments for further ovotransferrin extraction.

Our study indicates the applicability and suitability of CCD for efficient extraction of ovotransferrin from egg white. Central composite design takes into account interactions between the process variables. It is well known that presence of two or more components affect any bioprocess directly and individual components have profound effect on the results, but CCD takes one-step forward to quantify the effect of interaction of two or more components present as process variables. As the outcome of the process also depends on the interaction between the process components, statistical designs (e.g., CCD) prove to be more accurate, reliable, and precise in predicting the results than the traditional OFAT approach. Ovotransferrin has strong affinity towards iron, as it contains charged amino acid residues that bind to iron. Ionization of these residues is affected by variations in pH and this effect can be seen in the extraction runs, where pH has been varied keeping other parameters (ethanol and FeCl_3_) constant. From model equation (2), it is pretty evident that ethanol and pH have direct positive effect on the extraction process of ovotransferrin, while FeCl_3_ affects the process negatively. The negative effect of FeCl_3_ was overcome by the positive effect of ethanol and pH as the former was almost 9-10 times weaker than ethanol and pH or a combination thereof. The interactions between FeCl_3_, pH, and FeCl_3_ with ethanol promote higher recovery of ovotransferrin that in turn nullifies the negative effect of the interaction between ethanol and pH. The increase in pH alone enriches anions in the solution, positively affecting ovotransferrin extraction. The requirement for ethanol and volume of 25 mM FeCl_3_/50 mL of egg white increases when pH was taken to 7. When the pH was increased to 8, the requirement of ethanol as well as 25 mM FeCl_3_/50 mL of egg white decreased with the same levels of extraction. The increase in ethanol from 40 to 48% (v/v) showed negative effect on the recovery, at different volumes of 25 mM FeCl_3_/50 mL of egg white. Other statistical parameters that confirm the viability of the selected modified statistical model are coefficient of determination, lack of fit, and *p* values of the generated model and parameter interaction terms. The experiments showed a high value of determination coefficient (*R* ^2^) = 0.945 with an adjusted *R* ^2^ equal to 0.896. These values are high enough to confirm the accuracy of the predicted responses by the selected model. Apart from the interactions between ethanol and pH (*p* value = 0.8) and interaction of pH and 25 mM FeCl_3_ solution (*p* value = 0.97), all other major interactions were found to be above 95% confidence levels. The major parameters affecting the extraction of ovotransferrin were Var 3 (*Q*) with a *p* value of 0.0006 followed by Var 2 (*Q*) with a *p* value of 0.004 and Var 1 (*Q*) with a *p* value of 0.005. These terms are highly significant and affect the process in a positive manner. Total degree of freedom for the system was found to be 23 with 14 degrees of freedom for standard error (Table 3).

Similar observations for the interactions between pH and volume of FeCl_3_ have also been observed. FeCl_3_ solution results in a lower pH when solubilized in water. Therefore, it becomes necessary and also evident from the existing runs that a higher pH is suitable for suppressing the acidic nature of Cl^−^ ions released by solubilization of FeCl_3_ as well as for preventing ovotransferrin precipitation. Maximum productivity is achieved towards the center of the parameters and maintains it through increasing ethanol and FeCl_3_ concentration. Owing to the promising results and high predictability of the study, we can further develop the process at industrial scale. Many studies including animal and human trials are needed to establish ovotransferrin as a natural immune modulatory agent similar to lactoferrin. As the research progresses, more insights into the action of ovotransferrin would be gained that will open doors for new applications and opportunities. Developing process know-how early for ovotransferrin extraction would significantly reduce the downtime required for any molecule to move from lab to pilot stage and production scales.

In conclusion, this study suggests the applicability and suitability of central composite design and optimization techniques in designing of efficient extraction conditions for the major antimicrobial enzyme, that is, ovotransferrin from chicken egg white. 3D response surface plots and 2D plots for the effect of extraction conditions on enzyme yield enlighten us about interactive and individual effects of the extraction conditions on the enzyme yield. The applied method has ascendancy as it not only optimizes the extraction condition (for maximum ovotransferrin recovery) but also explains the effect of the independent variables (extraction conditions) on the dependent ones (the enzyme yield). Such information may be very useful in understanding and designing of the overall production process of the said antimicrobial metabolite/enzyme for commercial applications.

## Figures and Tables

**Table 1:** Design parameters for the statistical design.

Variables	Range selected for the study
Ethanol	31.5%–48.5% (v/v)
pH	6.3–9.7
Volume (mL) of 25 mM FeCl_3_/50 mL of egg white	0.08–0.92

**Table 2:** Design of experiments and ovotransferrin yield for each run.

S. number	Ethanol % (v/v)	pH	Volume (mL) of 25 mM FeCl_3_/50 mL of egg white	Ovotransferrin yield (mg/mL)
1	35.00	7.00	0.25	4.10
2	45.00	7.00	0.25	5.70
3	35.00	9.00	0.25	4.30
4	45.00	9.00	0.25	6.00
5	35.00	7.00	0.75	3.70
6	45.00	7.00	0.75	7.30
7	35.00	9.00	0.75	4.30
8	45.00	9.00	0.75	7.30
9	40.00	8.00	0.08	3.70
10	40.00	8.00	0.92	7.10
11	40.00	6.32	0.50	5.30
12	40.00	9.76	0.50	6.30
13	31.50	8.00	0.50	5.80
14	48.50	8.00	0.50	7.00
15	40.00	8.00	0.50	7.00
16	40.00	8.00	0.50	7.10
17	40.00	8.00	0.50	7.30
18	40.00	8.00	0.50	6.90
19	40.00	8.00	0.50	6.60
20	40.00	8.00	0.50	7.30
21	40.00	8.00	0.50	6.70
22	40.00	8.00	0.50	7.30
23	40.00	8.00	0.50	7.10
24	40.00	8.00	0.50	7.40

**Table 3:** ANOVA for ovotransferrin extraction. *R* ^2^ = 0.94553; Adj. *R* ^2^ = 0.89623.

	SS	df	MS	*p*
(1) Var 1 (L)	10.65997	1	10.65997	0.000188
Var 1 (Q)	1.79042	1	1.79042	0.048878
(2) Var 2 (L)	0.64872	1	0.64872	0.026131
Var 2 (Q)	4.74469	1	4.74469	0.004786
(3) Var 3 (L)	5.06342	1	5.06342	0.003841
Var 3 (Q)	8.04183	1	8.04183	0.000655
1L by 2L	0.02645	1	0.02645	0.080257
1L by 3L	1.44500	1	1.44500	0.045920
2L by 3L	0.00045	1	0.00045	0.974452
Error	2.92722	14	0.32337	
Total SS	38.37230	23		

**Table 4:** Regression analysis, Fischer value, and *p* value determinations. Regr. coefficients; Var 1: Var 3; *R* ^2^ = 0.94553; Adj. *R* ^2^ = 0.89623, 3 factors, 1 block, 24 runs; MS residual = 0.323726.

	Regression coefficient	*t*(14)	*p*
Mean/Interc.	−56.7598	−2.68155	0.017891
(1) Var 1 (L)	1.1429	2.99944	0.048532
Var 1 (Q)	−0.011	−3.05644	0.005878
(2) Var 2 (L)	9.0029	2.86487	0.012478
Var 2 (Q)	−0.5214	−3.34767	0.004786
(3) Var 3 (L)	−0.1206	−2.91122	0.051208
Var 3 (Q)	−11.2827	−4.35829	0.000655
1L by 2L	−0.0115	−0.24995	0.806257
1L by 3L	0.3400	2.94745	0.050940
2L by 3L	0.0300	0.03260	0.974452
